# Observations on the Use of Gelatine in Intermitting Fevers

**Published:** 1803-10-01

**Authors:** M. Gilbert


### OBSERVATIONS ON THE USE OF GELATINE IN INTERMITTING FEVERS; *by* M. GILBERT. *Read at the Medical Society of Paris, March* 18, 1803.

AN ingenious theory led M. Seguin to the medical use of gelatine; he thinks, that what he calls the febrile matter of intermitting fevers, is decomposed by the gelatine, in consequence of which he instituted many experiments, the result of which has confirmed him in this theory. The object of the author of this paper is to offer to the Society an observation which he followed through all its details, and which, he thinks, leaves no doubt whatever of the efficacy of this new remedy in the disease in question. At present a number of persons, nominated by the National Institute, are occupied at the Hospital of the Medical School, in researches on the administration and effects of gelatine, and the author wishes to second the efforts of the Institute by those observations which his particular practice has afforded. The author tells us, that he made his first trials at the military hospital of Val-de-Grâce, and he found that the immediate effect of taking the gelatine before the cold fit, was, to moderate in a remarkable manner its intensity, and to render the access in general more mild. He further observed, that it produced, in many persons, a diarrhœa more or less considerable; and here closed the author's enquiries. The difficulty of restraining military men in hospitals, the dislike to persevere a sufficiently long time in the use of medicine, and the want of exactness on the part of the patients in following his prescriptions, prevented him from offering to the Society, the facts which he had remarked in the hospital. He preferred to draw his conclusions from those cases which his private practice offered, as affording more certain and sure ground for observation.

The following case is intended as an illustration of the good effects of gelatine; A woman, aged forty-seven years, thin, and of a delicate constitution, and whose general appearance manifested an irregular digestion, and imperfect biliary motion, by profession a cook, was attacked in the month of September last, with a double tertian fever, which at the end of six weeks weakened her considerably; the shivering fits were long and violent, parching heat, an obvious biliary irritation; the access came on at the distance of twelve hours, repeated every day, but the fits were less strong every other day; such were the circumstances which led the author to fear the fever might change to a bilious remittent. The woman lived in the village of Sceaux, near Paris, where this fever wore an epidemical appearance, but seldom proved fatal. After emptying the stomach, the author administered the bark in substance during fifteen days, in consequence of which the fever disappeared, and the patient's health returned. Towards the end of February, the fever re-appeared but with tertian type. The paroxysms which were severe, were followed by great languor and general debility. During six weeks the disease resisted the general remedies, among which bitters formed a great part; the author could not venture to prescribe the bark, as well on account of the disgust of the patient to that remedy, as of considerable pains in the breast, of which the patient complained much, and which it was apprehended bark might increase. This was the state of the case when the author heard of the use of gelatine in this disease; he thought the opportunity a fair one for a trial, and which was done in the following manner. On the 17th of March, immediately before the cold fit, eighteen cakes or *tabletts* of the gelatine were taken in three doses, after the interval of ten minutes between each; each cake contained two drachms of the gelatine, and which was melted without any addition of water; the effect was immediate and astonishing, both the length and intensity of the paroxysms were diminished, the heat was more mild, and instead of twelve hours, the ordinary period of duration of the fit, it lasted but six. The regimen necessary to be observed, and which consists in a soup, an hour after the access, some roast meat, bread, prunes, and half a pint of wine, was strictly attended to. Three hours after the access, twelve drachms of the gelatine were administered; four hours after, fourteen more; two hours after meals, the same quantity. This was continued every day. The 19th the same was continued, and the access was found to come on two hours earlier; no shivering, the sense of chilliness trifling, the heat pretty strong, general lassitude, pain in the head, cholic pains after the second dose of the gelatine, and immediately after which a bilious purging came, so as to oblige the patient to go six times in the space of twenty minutes; at the end of four hours, the patient found herself well; the paroxysm lasted but three hours, consequently had diminished three-fourths. The 20th she felt herself comfortable, and began to occupy herself, which she had not done for some time; the gelatine was continued, no purging purging followed, and the face began to assume a florid healthy appearance. The 21st, the access came on two hours earlier; no shivering, gentle heat; the fit continued but an hour and a half; a diarrhœa came on as before. The 22d, intermediary day, the same regimen and use of the gelatine unattended by diarrhœa; the functions of digestion were much better, appetite more strong, and an obvious increase of powers; the patient employed herself as in a state of health. On being asked what sensation she experienced after taking the gelatine, she answered, that she felt a gentle heat internally. The 23d, the same treatment was continued; the access was hardly perceivable; the pain in the head and diarrhœa continued but half an hour. The 25th, there was no fever, no shivering, but a slight diarrhœa, and flying pains in the head. This day, which was the day of accession, the patient did her business as on the intermediate days. From that period to the present, 22d of April, the fever has not appeared. The use of the gelatine was persevered in fifteen days. After the disappearance of the symptoms, her strength, &c. were better than before she was attacked. Since the gelatine has been discontinued she complains of a weight in her stomach after meals, and which she did not experience during its use.

Such is the history of the case which M. Gilbert gives of the administration of the gelatine; if to this be added the number of observations made by M. Seguin, and which have been reported to the National Institute, as well as the corroborating testimony of the persons directed to pursue the inquiry in the hospital of the Medical School, we can hardly doubt but that the materia medica will be soon enriched by a new remedy.

The author asks, what notion we should form of the action of gelatine on the animal economy? The essential character of this substance, is that of being a matter highly nutritive and analeptic, that which has the least want of assimilation to be converted to our use, and changed to our proper substance; such is the point of view in which animal chemistry at present places it. This principle admitted as incontestible, let us, says the author, enquire into the nature of the fever; and without absolutely determining its proximate cause, let us judge of it after the inductions which the union of its remote causes present. It is an opinion generally received at the present day in physic, that intermitting fevers are produced by the action of a sedative cause, or of a debilitating one, which particularly affects the nervous system; and what proves this assertion is, that all those causes which debilitate, such as marshy exhalations, contagion, weakness of the organs of digestion; the passions which depress, as fear, &c. repeated purges, sudden changes of climate, produce and keep up intermittents; while, on the other hand, every cause which excites, whether physical or moral, bark, wine, opium, the return of the warm season, a sudden fit of dissipation, a vigorous effort of the imagination, &c. suspend or cure intermittent fever. From all this may we not conclude that gelatine produces on the stomach and the numerous nerves which go to that organ, an excitement which is propagated to all the system of the animal economy? And what seems to favour this opinion is, that in proportion as the fever is subdued, the powers return, the healthy appearance of the face begins to shew itself; in short, every thing announces that to the sedative cause which predominated during the fever, has succeeded an exciting action, which cured the disease; and what proves that the gelatine acts on the nervous system is, the suddenness of the effect produced on entering the stomach, an immediate diminution is perceivable in the degree of heat, and the spasmodic state of the skin; effects which are well known to be owing to nervous irritation.

The author tells us, that his intention was only to present a simple, theoretical view of the question, and which he believes to agree with the doctrine of M. Seguin, and which is supported by facts. M. Seguin, it appears, has proposed a new theory. The knowledge of the chemical action of gelatine on particular animal substances, when brought in contact, enables him to explain its mode of operation in the cure of intermitting fevers. Until M. Seguin brings the proofs of his doctrine, the author recommends to medical men the necessity of multiplying properly attested facts. He has imagined, that the Medical Society would gladly receive his communications on this subject; and to leave no difficulty in the way of those who may think the question worthy of further inquiry, he gives the process, which M. Seguin follows in the preparation of the gelatine,

| Mode of preparing Gelatine. | ℔ |
|---|---|
| Of the finest and most transparent glue — | 3 |
| Common water — — — — — | 15 |
| Melt and clarify it with white of eggs, and filter it through a cloth, to which add of sugar . | 3 |

Evaporate the whole on a slow fire, till the quantity of water be reduced to four-fifths; there will remain in the mixture equal quantities of the materials, sugar, gelatine, and water, 3lb. Such is the preparation in use. Run the liquor on a glass mould with raised edges, and about a square foot in surface; the substance must be left to coagulate, and which is afterwards to be dried on an iron grating, divided into spaces of an inch square; you will have cakes containing two drachms each dose. On the commencement of the shivering, from four to six of these cakes are to be given in three doses, at the interval of ten minutes; and during the intermittent, from an ounce and a half to two ounces are to be given every four or six hours.

